# GC–MS profiling of *Bacillus* spp. metabolites with an *in vitro* biological activity assessment and computational analysis of their impact on epithelial glioblastoma cancer genes

**DOI:** 10.3389/fchem.2023.1287599

**Published:** 2023-12-05

**Authors:** Muhammad Naveed, Huda Ishfaq, Shafique Ur Rehman, Aneela Javed, Muhammad Waseem, Syeda Izma Makhdoom, Tariq Aziz, Metab Alharbi, Abdulrahman Alshammari, Abdullah F. Alasmari

**Affiliations:** ^1^ Department of Biotechnology, Faculty of Life Sciences, University of Central Punjab, Lahore, Pakistan; ^2^ Department of Basic and Applied Chemistry, Faculty of Sciences, University of Central Punjab, Lahore, Pakistan; ^3^ Department of Healthcare Biotechnology, Atta-ur-Rahman School of Applied Biosciences (ASAB), National University of Science and Technology Islamabad, Islamabad, Pakistan; ^4^ Department of Agriculture University of Ioannina, Arta, Greece; ^5^ Department of Pharmacology and Toxicology, College of Pharmacy, King Saud University, Riyadh, Saudi Arabia

**Keywords:** *Bacillus* spp., bacterial metabolites, biological potential, epithelial glioblastoma cancer, PCR

## Abstract

**Background:** Bacterial metabolites play a crucial role in human health and have proven effective in treating various diseases. In this study, the 16S rRNA method and streaking were employed to isolate and molecularly identify a bacterial strain, with the goal of characterizing bioactive volatile metabolites extracted using nonpolar and polar solvents.

**Methods:** Gas chromatography–mass spectrometry (GC–MS) analysis was conducted to identify 29 compounds in the bacterial metabolites, including key compounds associated with *Bacillus* spp. The main compounds identified included 2-propanone, 4,4-ethylenedioxy-1-pentylamine, 1,2-benzenedicarboxylic acid, 1,1-butoxy-1-isobutoxy-butane, and 3,3-ethoxycarbonyl-5-hydroxytetrahydropyran-2-one.

**Results:** The literature indicates the diverse biological and pharmacological applications of these compounds. Different concentrations of the metabolites from Bacillus species were tested for biological activities, revealing significant inhibitory effects on anti-diabetic activity (84.66%), anti-inflammatory activity (99%), antioxidant activity (99.8%), and anti-hemolytic activity (90%). Disc diffusion method testing also demonstrated a noteworthy inhibitory effect against tested strains.

**Conclusion:** *In silico* screening revealed that 1,2-benzenedicarboxylic acid exhibited anticancer activity and promising drug-designing properties against epithelial glioblastoma cancer genes. The study highlights the potential of *Bacillus* spp. as a valuable target for drug research, emphasizing the significance of bacterial metabolites in the production of biological antibacterial agents.

## 1 Introduction

*Bacillus* species, ubiquitous in nature, are known for their remarkable metabolic versatility and have been extensively studied for their potential in the production of various biologically active compounds (Ajilogba and Babalola, 2019). Recent research has focused on identifying metabolites from *Bacillus* spp. and investigating their pharmacological properties, including their impact on cancer cells. Glioblastoma is one of the most aggressive forms of brain cancer, characterized by rapid proliferation and resistance to therapy (Valli et al., 2012). The use of natural compounds and their derivatives to combat this devastating disease has gained significant attention. This study explores the potential of *Bacillus* species metabolites in inhibiting glioblastoma cancer cell growth through *in vitro* experiments while also computationally assessing their effects on epithelial glioblastoma cancer genes.

In the realm of natural product discovery, gas chromatography–mass spectrometry (GC–MS) has emerged as a powerful tool for identification and analysis of diverse chemical compounds in microbial extracts (Faridha Begum et al., 2016). The application of GC–MS to *Bacillus* spp. extracts allows for the identification of a wide array of metabolites, which can be further assessed for their biological activities (Caulier et al., 2019). This analytical technique provides invaluable information on the composition of metabolites produced by *Bacillus* spp., laying the foundation for understanding their potential pharmacological properties (Karlovsky, 2008).

*In vitro* biological studies form a critical part of this research. The goal is to assess the biological activities of *Bacillus* spp. metabolites, particularly their impact on glioblastoma cancer cells (Motta et al., 2008). The *in vitro* experiments will provide insights into the cytotoxicity and anticancer potential of these metabolites, shedding light on their ability to inhibit glioblastoma cell growth and potentially induce apoptosis. These experiments will be conducted following established protocols and methodologies to ensure the robustness of the results (Mishra et al., 2015).

Complementary to *in vitro* studies, computational analysis is employed to investigate the effects of *Bacillus* spp. metabolites on epithelial glioblastoma cancer genes (Mora et al., 2015). By using advanced bioinformatics tools and databases, this approach will allow us to explore how these metabolites may influence the expression and regulation of genes associated with glioblastoma (Zugazagoitia et al., 2016). By integrating experimental and computational data, we aim to gain a comprehensive understanding of the potential therapeutic value of *Bacillus* spp. metabolites in glioblastoma treatment (Mohan et al., 2021).

This study aims to unveil the potential of *Bacillus* spp. metabolites as a source of natural compounds with the ability to inhibit glioblastoma cancer cell growth and to computationally elucidate their impact on epithelial glioblastoma cancer genes. The integration of GC–MS analysis, *in vitro* biological experiments, and computational methods will provide a holistic approach for exploring the pharmacological properties of these metabolites and their therapeutic potential in the context of glioblastoma treatment.

## 2 Materials and methods

### 2.1 Bacterial strain isolation and identification

Bacteria are isolated from a soil sample which was obtained from Punjab University botanical garden. Serial dilutions of the soil sample were prepared and spread on Luria Broth (LB) agar plates. The plates were allowed to solidify before serial dilutions of the sample were inoculated and kept in an incubator at 30 °C for a 24-h growth period. A sterile loop was used to isolate colonies from bacterial cultures, which were then streaked onto new plates for further investigation.

### 2.2 Molecular identification of bacterial strains

Molecular identification of bacterial isolates will be accomplished through DNA extraction and PCR testing of their DNA isolates. For this, the cetyltrimethylammonium bromide (CTAB) technique was used to distinguish between bacterial strains as it is soluble in both alcohol and water.

#### 2.2.1 DNA extraction method

DNA extraction from bacterial cells followed the CTAB method. Initially, 1.5 mL of inoculated LB was centrifuged at 15,000 rpm for 5 min, and the resulting pellet was discarded. The pellet was dissolved in 570 µL of TE buffer, combined with 2–3 µL of proteinase K and 30 µL of 10% SDS, and incubated at 37 °C for 1 h. Post-incubation, 100 µL of 5 M NaCl and approximately 80 µL of CTAB were added, and the solution was incubated at 37°C for 30 min. Then, 500 µL of chloroform and isoamyl alcohol (24:1 ratio) were added, followed by centrifugation at 13,000 rpm for 5 min. The supernatant was transferred to another Eppendorf tube and re-centrifuged for 6 min with phenol, chloroform, and isoamyl alcohol (25:24:1 ratio, respectively). The aqueous layer was removed, cold ethanol was added, and the mixture was stored at −20 to −80 °C for approximately 1 h. Following cold storage, the Eppendorf tube was centrifuged again for 12 min at 13,000 rpm, and all liquids were drained before air-drying with a fan. Subsequently, 20–30 µL of TE buffer was added, along with 2 µL of RNase for RNA removal, followed by a 30-min incubation at 35°C in a water bath. To this solution, approximately 150 µL of ethanol and 25–30 µL of TE buffer were added, mixed, and centrifuged for 12 min at 13,000 rpm to remove the solution. Finally, the pellets were air-dried in the Eppendorf tube and then suspended in 45–50 µL of TE buffer.

#### 2.2.2 Gel electrophoresis

For DNA analysis, a 1.5% agarose gel was prepared and placed in an electrophoresis chamber. Running buffer (1X TAE) was added to fully submerge the gel (2–3 mm above the gel). Next, 3 µL of 6X DNA loading dye (Thermo Scientific) was mixed with 5 µL of the extracted DNA samples. Each well was loaded with a total volume of 8 µL, containing the prepared DNA. After loading all the samples, the electrophoresis chamber was sealed, and electrodes were connected. The gel was run at 100 V for 30 min. Upon completion, the gel was carefully removed from the tray using gloves and evaluated under UV light in the gel doc to determine the presence of DNA.

#### 2.2.3 PCR

In the PCR reaction procedure, all necessary reagents were combined in autoclaved PCR tubes. Initially, 12.5 mL of the master mix was added to each tube, followed by the addition of 2 µL of the DNA sample and 8.5 µL of water. Subsequently, the forward and reverse primers were individually introduced into the tubes, and the components were gently vortexed to ensure thorough mixing. Then, the prepared sample reactions were then placed in the PCR machine. For the amplification of 16S rRNA, a temperature gradient was applied within a thermocycler under ideal reaction conditions. Universal primers (27F/1492R) were employed to amplify unidentified bacterial strains, targeting the conserved 16S rRNA gene sequence. The PCR reaction amplification conditions involved an initial denaturation at 95°C for 5 min, followed by denaturation at 95°C for 1 min, annealing at 54°C for 30 s, extension at 72°C for 1 min, and a final extension at 72°C.

### 2.3 Bacterial metabolite extraction and identification

#### 2.3.1 Bacterial extract preparation

A bacterial isolate was subjected to a series of incubation and fermentation processes in a 500-mL Erlenmeyer flask. To create the broth, a mixture of tryptone, NaCl, and yeast extract was prepared and added to the flask. Subsequently, the flask was placed inside a shaking incubator for a duration of 3–4 days. Following this initial incubation, the fermentation media comprising tryptone (5 g), NaCl (5 g), oatmeal (0.5 g), phenol red (0.09 g), and distilled water (500 mL) were added to the same flask, and the entire setup was further incubated at 37°C while being shaken at 110 rpm.

#### 2.3.2 Separation of compounds from bacterial metabolites

After the completion of the fermentation process, the culture broth was divided into three separate funnels, with each funnel containing 500 mL of the broth. To investigate the efficacy of different extraction solvents, namely, ethyl acetate, methanol, ethanol, and n-butanol, they were added individually to the funnels. This allowed for a comparative analysis of their ability to extract specific compounds or substances from the culture broth. Overnight, the solvents and the culture broth in the separating funnels formed distinct layers, indicating successful extraction of different compounds from the broth into each solvent. These solvent layers were carefully collected in separate beakers to ensure proper isolation of the extracted materials.

To remove any remaining traces of the solvents and to concentrate the extracted compounds, the beakers were placed in a water bath set at 60°C for drying. This step aimed to evaporate the solvents, leaving behind the concentrated extracts from the culture broth. Once the extracts were fully dried and free of any residual solvents, they were transferred into test vials for further analysis and experimentation. The use of test vials allowed for convenient and organized storage of the extracts, ensuring they were readily available for subsequent research and characterization.

#### 2.3.3 Gas chromatography–Mass spectrophotometry

The crude sample was sent for size- and polarity-based GC-MS analysis. The chemicals found in metabolites were identified using GC-MS. The GC-MS analysis was performed using a PerkinElmer GC Clarus 500 system and a gas chromatograph connected to a mass spectrometer. For GC-MS detection, an electron ionization system with an ionization energy of 70 eV was used in the electron impact mode. An injection volume of 2 µL was employed, and helium gas (99.999%) was used as a carrier gas at a continuous flow rate of 1°mL/min. The injector temperature was kept constant at 250°C, the ion-source temperature was kept constant at 200°C, and the oven temperature was programmed to begin at 110°C, then increased by 10 °C/min to 200°C, and then increased by 5 °C/min to 280°C, finishing with a 9-min isothermal at 280 °C. Fragments ranging in size from 45 to 450 kDa were used in the mass spectra. The total GC–MS run time was 36 min, with a solvent delay of 0–2 min. The relative percentage quantity of each component was determined by comparing the average peak area of each component to the total areas.

### 2.4 Biological activities

#### 2.4.1 Antioxidant analysis

By using the colorimetric DPPH (2, 2-diphenyl-1-picrylhydrazyl) test, the antioxidant capacity of the samples was determined *in vitro*. The technique was finished by estimating the DPPH solution’s inhibitory concentrations, scavenging activity percentages, and reduction in violet hue using the samples under investigation (El-Amier et al., 2023). For this experiment, 1 mL of 90 M DPPH was combined with various fractions of extracted bacterial metabolites, i.e., 150, 250, 350, 450, and 550 µL in the dark. Ascorbic acid was utilized as the standard at the same concentration of 150, 250, 350, 450, and 550 µL. At 37°C, the mixture was incubated for 30 min. The absorbance at 517 nm was measured using an ELISA plate, and the activity was expressed as a percentage inhibition.

$$\%\text{ Radical scavenging}=\text{Abs control }–\;\left(\text{Abs sample}/\text{Abs control}\right)\times 100.$$

#### 2.4.2 Anti-diabetic activity

Alpha-amylase test results were calculated using the anti-diabetic activity. The bacterial samples were used at various concentrations (200, 400, 600, 800, and 1000 µL), and metformin was used as the control. In the Falcon tube, an alpha-amylase solution was prepared. Test tubes were filled with various bacterial sample concentrations and 10 µL of alpha-amylase and placed in an incubator set at 37 °C. Then, the same tubes were filled with 50 µL of a 1% starch solution and left to stand for 1 h. A 50-µL solution of 1% iodine was added and left to incubate for 30 min after the first hour. An ELISA reader set to 630 nm was used to check the absorbance at the end. The following equation was used to determine anti-diabetic activity percentages:

$$\%\text{ Inhibition}=\left[\left(\text{Abs control }-\text{ Abs sample}\right)\right]/\text{Abs sample}\times 100.$$

#### 2.4.3 Anti-inflammatory activity

The protein denaturation bioassay was used to assess the potential for anti-inflammatory affects *in vitro* (Chaudhari et al., 2013). These included 100, 200, 300, 400, and 500 µL of different bacterial sample concentrations. Aspirin was utilized as a control in this analysis. The above concentrations were combined in test tubes marked with the corresponding concentrations with 2.8 mL of the PBS solution and 0.2 mL of fresh egg white, added to a water bath having a temperature of 37°C, and heated for 20 min. After the incubation, they were placed in the water bath at 70°C for 5 minutes.

% Inhibition of protein denaturation = 1 – Abs of Sample/Abs of Control X 100.

#### 2.4.4 Antibacterial activity

The antibacterial effectiveness of various strains such as *Serratia marcescens* and *Staphylococcus aureus* was measured using the disc diffusion method. The nutrient agar was mixed with 200 mL of distilled water in a conical flask (250 mL). The mixture was autoclave-sterilized for 15 min at 15 lbs and 121°C. The medium was allowed to cool to 45°–50°C. The mixture was shaken before being poured into sterile Petri dishes. After pouring, it was left for 15 min to solidify. The antibacterial activity of the bacterial samples was assessed using a disc diffusion assay with an inoculum of 106 bacterial cells/mL spread on nutrient agar plates. The bacterial sample was immersed in the antibiotic disc and sterilized filter paper until saturation. The discs were placed on top of agar plates that had been seeded with specific bacterial microorganisms. At 37°C, the plates were incubated for 18–24 h. The coloration around the disc was calculated and compared to that of the positive control after 18–24 h.

#### 2.4.5 Hemolytic activity

Five milliliter of human blood was drawn from a healthy person and placed in EDTA vials. The supernatant from centrifugation of blood was discarded. The pellet was rinsed with 150 mM NaCl, two–three times. By increasing the volume of blood suspension in phosphate-buffered saline (PBS) refrigerated at 4°C, the erythrocyte suspension was created. Several concentrations (50,100,150, 200, and 250 µL) of bacterial samples were mixed with 0.2 mL of the blood suspension in a micro-centrifuge tube. Following a 15-minute period of centrifugation at 16,000 rpm, a 30-minute incubation at 37°C was carried out. Following this, 100 µL of the supernatant was collected and combined with 900 µL of PBS to calculate the absorbance at 630 nm in the ELISA plate. PBS was used as a negative control, and 0.1% Triton X was used as a positive control.

The formula mentioned below was used to calculate hemolytic percentages:

% hemolysis=Abs of sample – Abs of –ve control / Abs of control X 100.

#### 2.4.6 Cytotoxicity analysis

U87 cells derived from human malignant glioblastoma multiforme were cultured for cell line activity in Dulbecco’s modified Eagle medium (DMEM)—high glucose with 10% FBS (Thermo Fisher Scientific, Waltham, MA, United States) and 1% pen–strep MTT (3-[4,5-dimethylthiazol-2-yl]-2,5-diphenyltetrazolium bromide (Sigma-Aldrich, St. Louis, MO, United States). The cell line studies of tested compounds (compound F) were carried out on these cells. The percentage cytotoxicity of all compounds was evaluated by dose-dependent MTT analysis. Exponentially growing cells were counted, and 10,000 cells per well were plated, in triplicates, in flat-bottomed 96-well plates (Nunc, Roskilde, Denmark).

The volume of the cells was kept at 100 μL per well. Each tested compound was dissolved separately in supplemented media to obtain different concentrations (200 ug/mL, 150 μg/mL, 100 ug/mL, 80 ug/mL, and 40 ug/mL). Each concentration of the drugs was added to the 96-well plate to obtain a final volume of ∼200 μL/well. In addition, each concentration was tested in triplicate on U87 cells. Control wells contained solvent control (without drug) and blank media (without cells). Subsequently, 5 mg/mL of MTT was dissolved in 1 mL PBS. Accordingly, 15 μL of the prepared MTT solution was added to each well and incubated for 3 h at 37°C, making intracellular purple formazan crystals visible under a microscope. Following the formation of formazan crystals, all of the solution from each well was removed. Then, a solubilizing solution, i.e., 150 μL DMSO, was added in each well. The plates were left at room temperature for a few minutes, while DMSO solution was mixed thoroughly by pipetting up and down to dissolve the formazan crystals. Finally, the absorbance of the cells was measured by using a spectrophotometer at 550 nm.

### 2.5 Computational analysis

#### 2.5.1 Retrieval of the epithelial glioblastoma-causing gene

Epithelial glioblastoma cancer genes were found using the human gene database (GeneCards, https://www.genecards.org/, retrieved on 10 July 2023). This database offers detailed and approachable information on all predicted and annotated human genes in a searchable format.

#### 2.5.2 Structural retrieval of bacterial metabolites

The PubChem database (National Institutes of Health (NIH)) is a free resource for obtaining chemical information. It includes data on the physical and chemical properties, chemical compositions, toxicity, biological activities, and health consequences of all small molecules or macromolecules that have been chemically altered. Safety and patentability were also addressed, and it was accessed on 13 July 2023.

#### 2.5.3 Functional annotation of retrieved genes

A set of tools known as g: Profiler (https://biit.cs.ut.ee/gprofiler) is frequently used in the standard pipelines of computational analysis focused on biological entities (genes and proteins). It carries out the functional enrichment analysis of genes (Raudvere et al., 2019) and was accessed on 14 July 2023.

#### 2.5.4 Protein–protein network construction (STRING)

STRING (https://string-db.org, version 11.0), accessed on 16 July 2023, imported 20 target genes from the Venn diagram intersection in order to methodically construct a PPI network to understand protein interactions. The PPI network was built using *homo sapiens*, with a medium confidence interaction score of 0.40 and the exclusion of any protein nodes that were not connected to anything else.

#### 2.5.5 Venn analysis

On 15 July 2023, the Venn diagram (bioinformatics.psb.ugent.be/webtools/Venn) was accessed to show how evolutionary genomics and bioinformatics systems are employed to develop drug and disease targets that intersect.

#### 2.5.6 Molecular docking analysis (PyRx)

Docking was done using PyRx and the PDB-sourced 3D structures of epithelial glioblastoma cancer genes. The compounds isolated from *Bacillus* spp. bacterial metabolites were docked with the hit molecule discovered through virtual screening. PubChem (https://pubchem.ncbi.nlm.nih.gov) was accessed to retrieve all of the compound structures in the SDF format. Of the compounds, 1,2-benzenedicarboxylic acid (PubChem: 33934) displayed the highest binding energies with all 20 epithelial glioblastoma cancer genes. The findings were displayed in terms of energies, and the substances considered the top candidates of anti-inflammatory activity had the least docking energies.

#### 2.5.7 QSAR modeling (3D-QSAR)

The cloud 3D-QSAR server can be accessed at http://chemyang.ccnu.edu.cn/ccb/server/cloud3dQSAR/. The website will run 3D-QSAR jobs by uploading IC50 and molecular structure values on 1 August 2022. Energy is reduced after molecules are transformed into 3D structures. They are all subjected to 3D-QSAR modeling after being randomly split into test and training sets. Other results are analyzed and sorted automatically, and force field files are automatically generated.

#### 2.5.8 Molecular dynamic simulation

It is a computer-aided simulation method for analyzing atoms or molecules’ physical motions. A few significant interactions can be identified using the molecular dynamics (MD) simulation of hydrogen bond interactions. MD simulations make virtual screening and protein docking possible. The IMODS server was used to run molecular dynamics simulations for this study. This server offers details on routes involving macromolecules or homology that can be searched using normal mode analyses.

#### 2.5.9 Pharmacokinetic ADME evaluation

A molecule’s water solubility, blood–brain permeability, hepatotoxicity, and physiochemical, pharmacokinetic, and medicinal chemistry were all determined using the free online tool SwissADME. SwissADME was employed to characterize the top compounds determined by docking results and having the highest inhibitory affinities for epithelial glioblastoma genes. It established the compounds’ toxicity and characteristics.

## 3 Results

### 3.1 Isolation and identification of bacterial strains

Isolation of the pure strain of *Bacillus* spp. was performed using the streaking method. The bacterial strains were streaked from the samples using LB medium. The collected bacterial samples were streaked on nutrient agar Petri plates in an aseptic environment in the biosafety cabinet. Bacterial growth and results were observed on the Petri plates.

### 3.2 Molecular characterization

**FIGURE 1:**
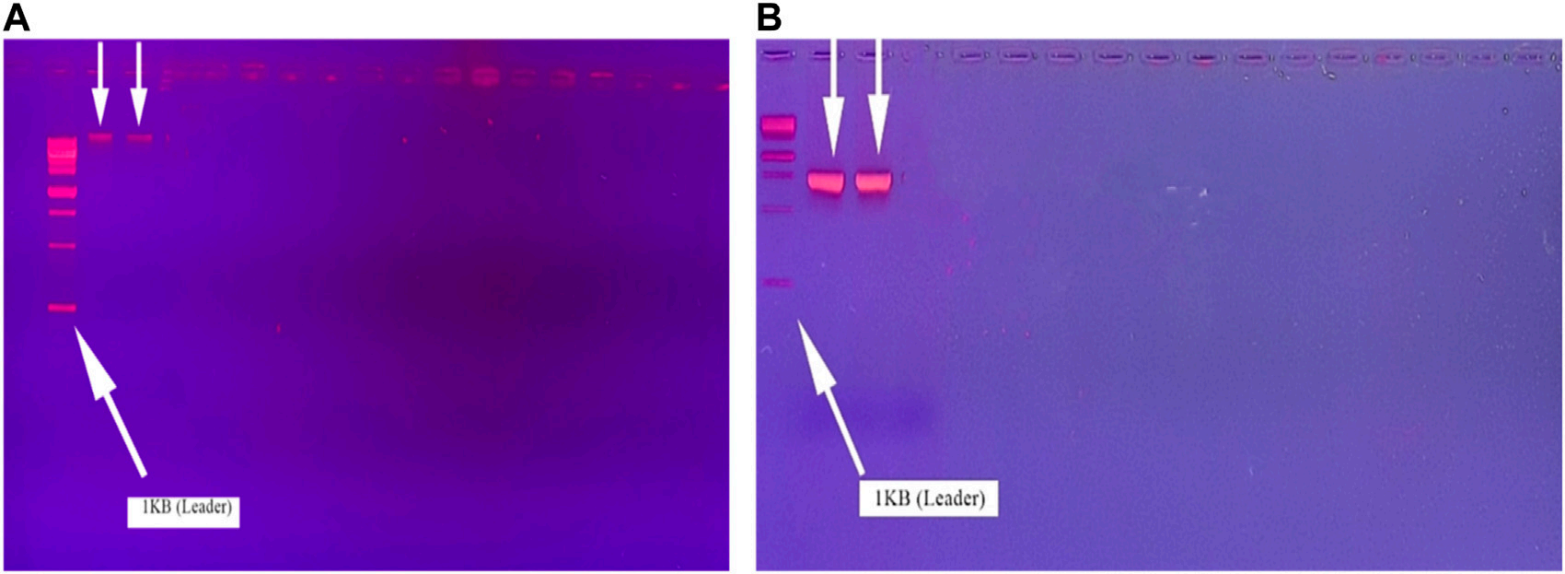
Molecular characterization. **(A)** Bacterial DNA extraction; **(B)** 16S rRNA amplification of *bacillus* spp. on gel electrophoresis.

Using the 10% CTAB method, DNA was extracted. The next step was to perform gel electrophoresis using 1.5% agarose gel. The DNA band is seen in Figure 4.2 after the DNA samples were run on the gel and visualized using the gel dock (Figure 1A). The 16S RNA region was amplified with 16S rRNA universal primers. The 16S RNA was amplified on 2% gel electrophoresis. After their confirmation in the gel documentation system, the thermocycler PCR (Applied Biosystems) was used to amplify the bacterial DNA extract. Using a set of 27F/1492R primers, 16S rRNA was amplified. The results of the amplified 16S rRNA are shown in Figure 1B.

### 3.3 Sequencing of 16S rRNA

**FIGURE 2:**
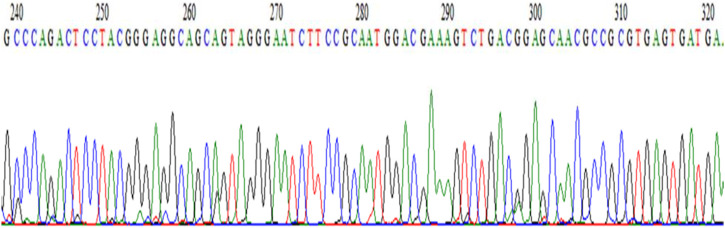
Chromatograph of the *Bacillus*.

The samples were delivered to a Malaysian company for sequencing after the visualization of 16S rRNA via gel electrophoresis. Sanger sequencing was carried out on the samples. The chromatograph’s results are shown in Figure 2.

### 3.4 Identification of 16S rRNA

The sequence obtained after sequencing was bio-edited and then identified using NCBI BLASTN. The results are displayed in Table 1.

**TABLE 1:** 16S rRNA BLASTN results.

Strain ID	Strain name	Number of nucleotides of the 16S rRNA gene	Sequence similarity (%)of the 16S rRNA gene with closely related taxa	Sequence query coverage (%)
MBBL2	*Bacillus* spp.	942 bp	93.79%	99%

### 3.5 Bacterial metabolite extraction and identification

#### 3.5.1 GC-MS results of the crude sample

A total of four solvents (n-butanol, methanol, ethyl acetate, and ethanol) were used. A total of 35 compounds from n-butanol ethyl acetate, methanol, and ethanol were identified, which are shown in Table 2.

**TABLE 2:** Compounds from GC-MS analysis of *Bacillus* spp.

Sr. No	Compound	Molecular weight	Molecular formula	Retention time
1	1,2-Benzenedicarboxylic acid	390	C_24_H_38_O_4_	17.80
2	1-Butoxy-1-isobutoxy-butane	202	C_12_H_26_O_2_	7.39
3	2-Propanone	202	C_11_H_22_O_3_	7.39
4	3-Ethoxycarbonyl-5-hydroxytetrahydropyran-2-one	188	C_8_H_12_O_5_	8.31
5	4,4-Ethylenedioxy-1-pentylamine	145	C_7_H_15_NO_2_	8.10
6	Allyl nonanoate	198	C_12_H_22_O_2_	16.45
7	Benzene	106	C_8_H_10_	3.90
8	Butane, 1,1-dibutoxy	202	C_12_H_26_O_2_	7.39
9	Decanoic acid	212	C_13_H_24_O_2_	16.45
10	di-tert-butyl decarbonate	218	C_10_H_18_O_5_	7.39
11	2,2'-[1,2 ethanediylbis (oxy)]bis-, diacetate	234	C_10_H_18_O_6_	8.10
12	Ethanol,2,2′-oxybis-, diacetate	190	C_8_H_14_O_5_	7.69
13	Hexadecane	594	C_40_H_82_O_2_	16.45
14	Octadecanoic acid	324	C_21_H_40_O_2_	16.45
15	o-Xylene	106	C_8_H_10_	3.90
16	Propanedioic acid	230	C_11_H_18_O_5_	7.39
17	p-Xylene	106	C_8_H_10_	4.28
18	1,3,5-Trisilacyclohexane	132	C_3_H_12_Si_3_	17.43
19	1,3-Dioxolane	116	C_7_H_14_O_2_	8.29
20	5-Hexen-3-ol	142	C_9_H_18_O	8.29
21	6-Ethyl-3- trimethylsilyloxydecane	258	C_15_H_34_OSi	18.62
22	Di-n-octyl phthalate	390	C_24_H_38_O_4_	17.80
23	Bis(2-ethylhexyl) phthalate	390	C_24_H_38_O_4_	17.81
24	Mono(2-ethylhexyl) ester	278	C_16_H_22_O_4_	17.81
25	2-Pentanone	116	C_11_H_22_O_3_	3.50
26	Decane, 1-fluoro	160	C_10_H_21_F	16.45
27	Ethylbenzene	106	C_8_H_10_	3.74
28	p-Dioxane	232	C_12_H_24_O_4_	7.39
29	Propanedioic acid	230	C_11_H_18_O_5_	7.39

### 3.6 Biological activities

#### 3.6.1 Antioxidant activity analysis

**FIGURE 3:**
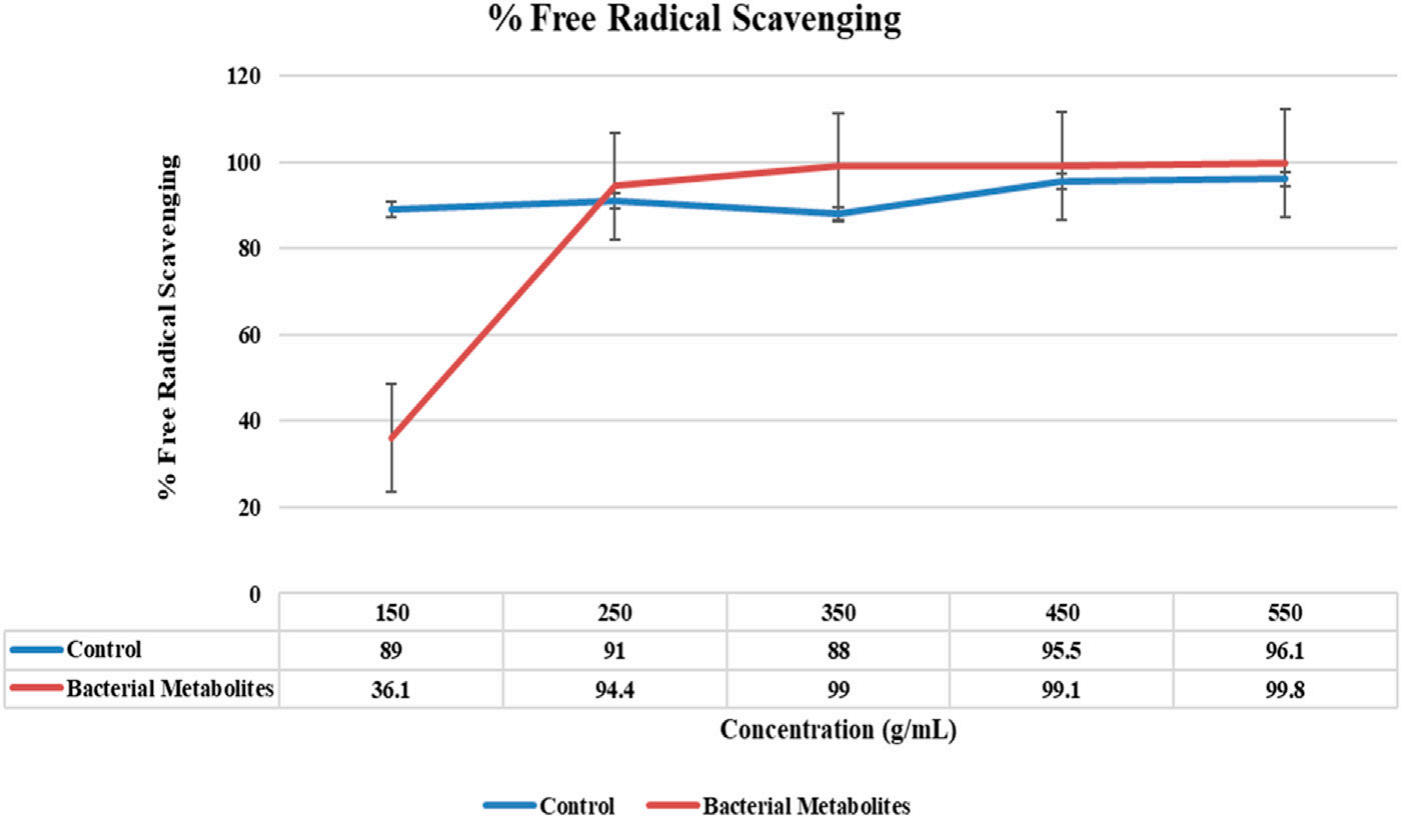
Graph for antioxidant activity showing the highest percent free radical scavenging of bacterial metabolites (99.8%) at a concentration of 500 μg/mL.

The DPPH radical’s ability to reduce was measured by the decline in its absorbance at 517 nm brought on by a number of antioxidants, demonstrating the link between antioxidants and radical scavenging. This decline in the absorbance of the DPPH radical is what triggers radical scavenging via hydrogen donation. At a maximum concentration of 500 g/mL, the bacterial metabolites exhibited the highest antioxidant activity of 99.8% (Figure 3).

#### 3.6.2 Peptide hemolytic activity

**FIGURE 4:**
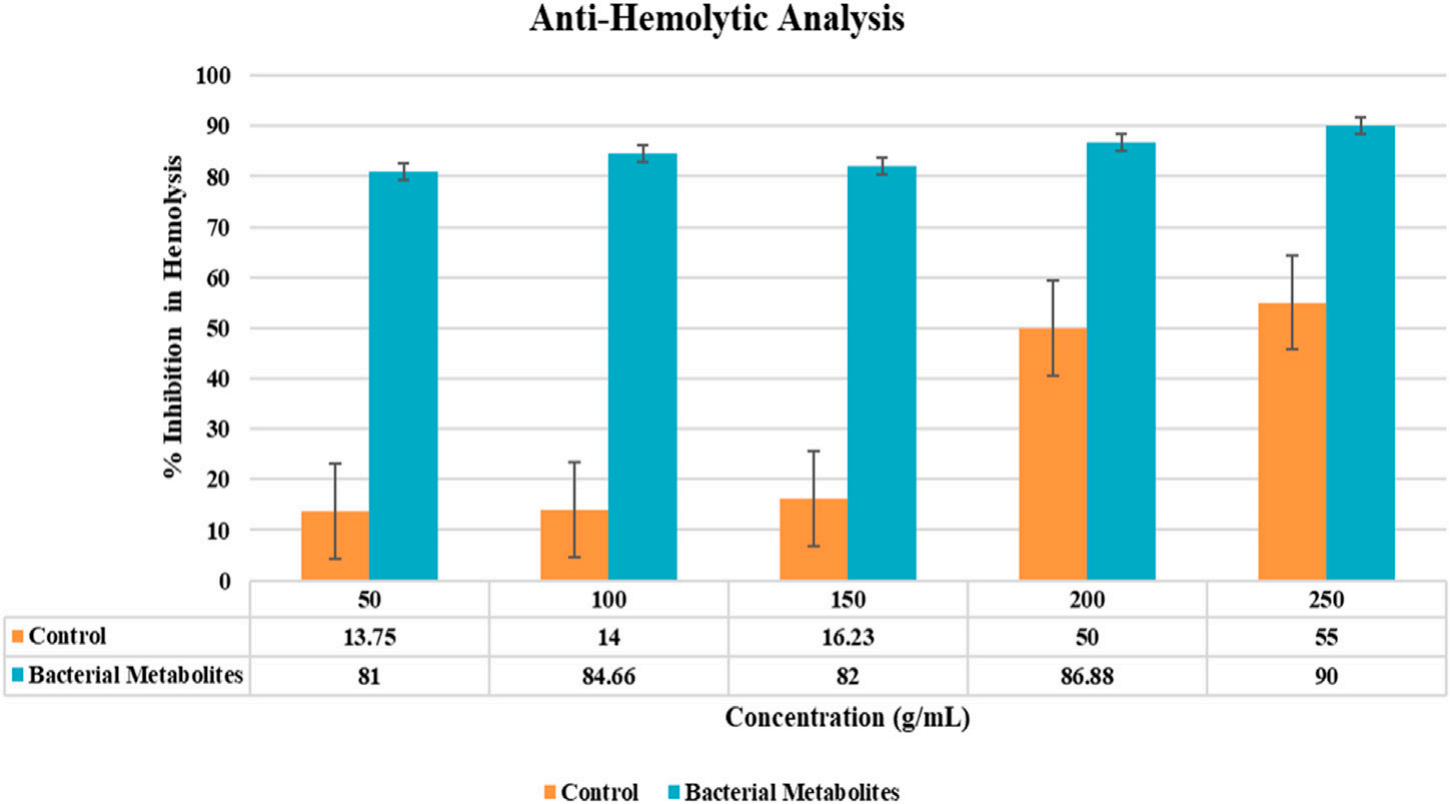
Graphical representation of activity showing % inhibition in hemolysis by bacterial metabolites at different concentrations.

The activity was concentration-dependent, and the results were shown after the hemolytic activity was completed. The highest hemolysis inhibition percentage was found to be 90% at the maximum concentration of 250 g/mL. Based on the percentage of hemolysis activity, the results showed that bacteria were non-toxic and biocompatible. Without assessing hemolysis activity, the use of bacterial metabolites under uncontrolled conditions is harmful (Figure 4).

#### 3.6.3 Anti-inflammatory activity

**FIGURE 5:**
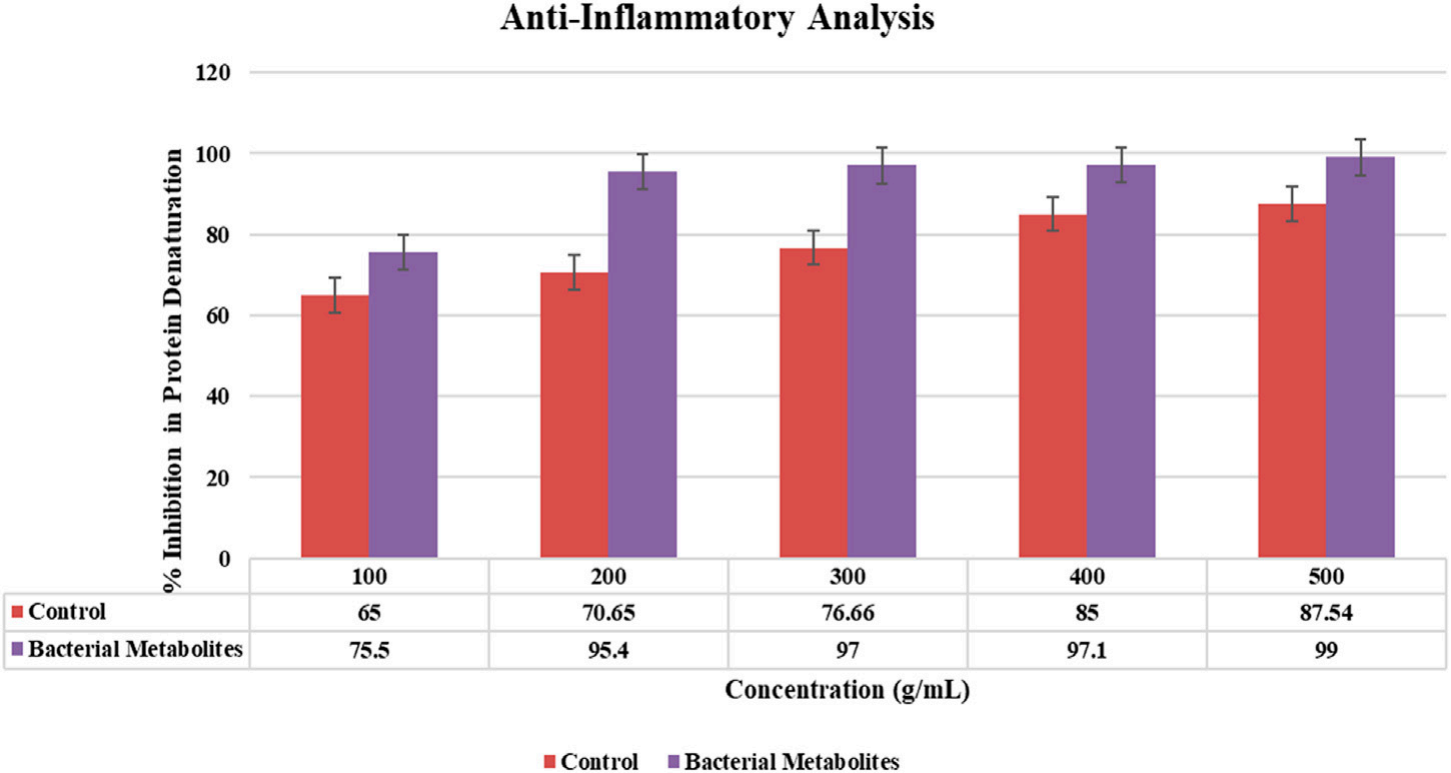
Graphical representation of % inhibition in protein denaturation showing 99% anti-inflammatory activity by bacterial metabolites at 500 μg/mL.

At various concentrations, the potential of bacterial metabolite extracts to protect denaturation of bovine serum albumin has been measured. At 500 g/mL concentrations of bacterial metabolites, the maximum percentage inhibition of protein denaturation was 99% (Figure 5). According to the findings, the bacterial metabolite is more effective than the control drug (aspirin) in inhibiting albumin protein denaturation.

#### 3.6.4 Anti-diabetic activity

**FIGURE 6:**
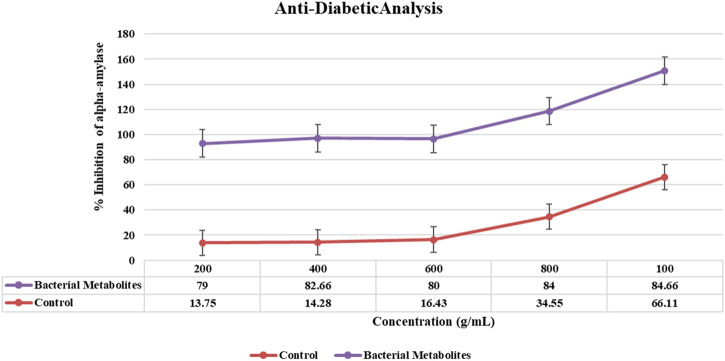
Graphical representation of anti-diabetic activity showing the % inhibition of α-amylase by bacterial metabolites at different concentrations.

Bacterial metabolites were identified as alpha-amylase enzyme inhibitors by use of the alpha-amylase assay because the intensity of red color was less. The percentage of inhibition increased with the concentration (200–1,000 g/mL; 79%–84.66%) and was dose-dependent. Bacterial metabolites exhibit a maximum inhibition of 84.66%, whereas metformin, a commonly used drug, only exhibits a maximum inhibition of 66.11% at the same dose (1,000 g/mL) (Figure 6).

#### 3.6.5 Anti-microbial activity

**FIGURE 7:**
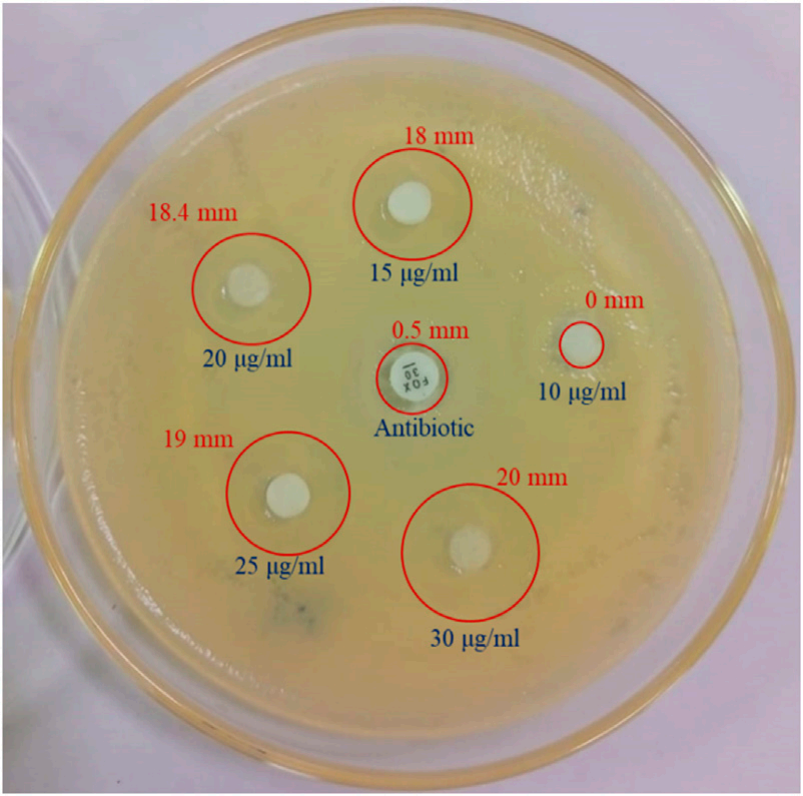
Anti-bacterial analysis of bacterial metabolites showing the zone of inhibition against cefoxitin antibiotic and bacterial metabolites at various concentrations.

The antibiotic cefoxitin was used to research the antimicrobial properties of bacterial metabolites. When compared to antibiotics, bacterial metabolites at 30 μg/mL demonstrated a minor zone of inhibition against *Serratia marcesens* at a 20 mm depth (Figure 7).

#### 3.6.6 Cytotoxicity analysis

**FIGURE 8:**
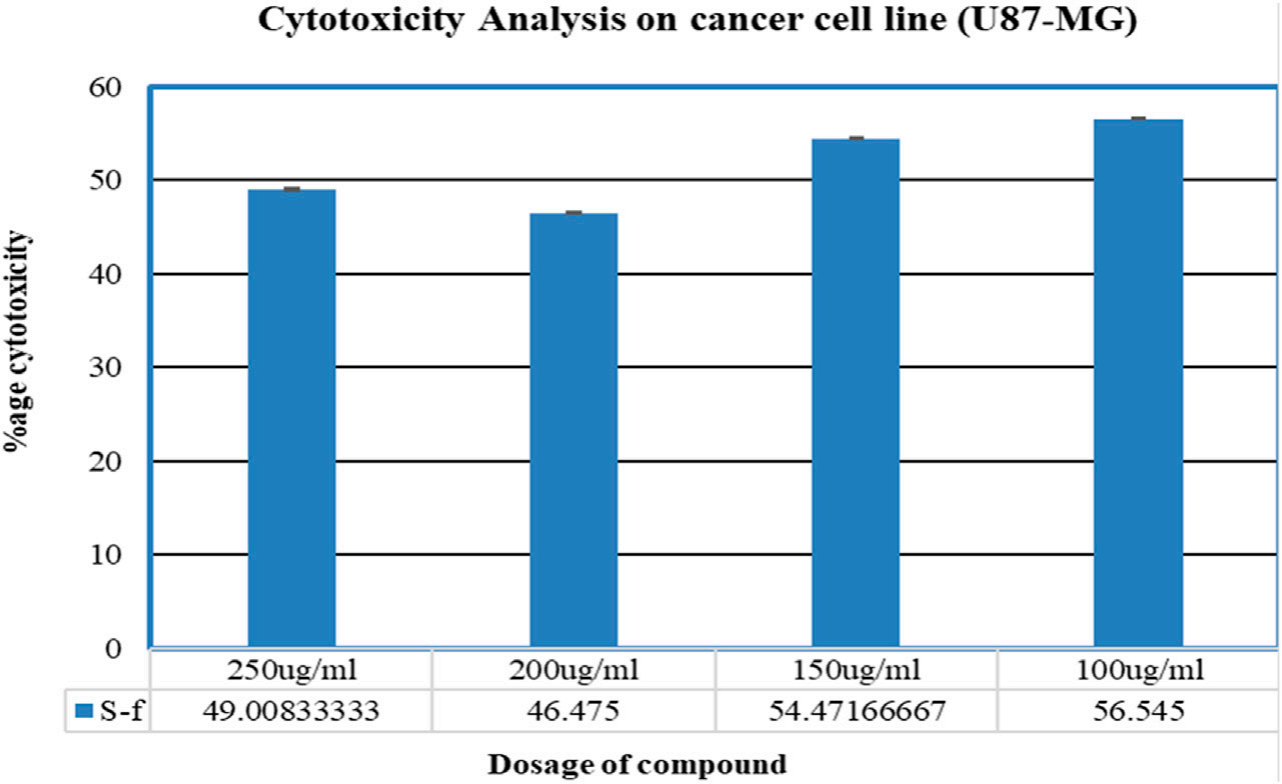
Graphical representation of % cytotoxicity of the U87-MG cell line and morphology of the U87-MG cell line after 24 h and comparison with the control.

The results obtained from the MTT analysis for the U87 cell line indicated that % cell cytotoxicity for “compound F” was 49% (250 ug/mL), 46.4% (200 ug/mL), 54.4% (150 ug/mL), 56.5% (100 ug/mL), and −5.8% (50 ug/mL). Therefore, It is evident from the results that compound “F” showed a significant trend in % cell cytotoxicity in a dose-dependent manner, i.e., with increasing concentration, cytotoxicity is also increasing (Figure 8).

### 3.7 Computational analysis

#### 3.7.1 Retrieval of epithelial glioblastoma cancer genes

GeneCards was used to search for the target genes associated with epithelial glioblastoma; there are 246 genes in GeneCards, with no overlapping of target genes. From GeneCards, a total of 20 genes associated with epithelial glioblastoma cancer were discovered. The genes were chosen based on prior studies that were significant in terms of drug development.

#### 3.7.2 Tertiary structure retrieval of genes (PDB)

The PDB was used to retrieve the complete gene sequences for 20 cancerous genes related to epithelial glioblastoma with the subsequent PDB ID: TP53 (1A1U), PTEN (1D5R), MSH6 (2GFU), APC (1BEB), CHEK2 (1GXC), PMS2 (1EA6), KRAS (1D8D), BR1P1 (1T15), CDKN2A (1A5E), MET (1FYR), EPCAM (4MZY), CTNNB (1G3J), BRAF (1UWH), MSH2 (208B), BARD1 (1JM7), RB1 (1AD6), MUTYH (1X51), TERT (2BCK), HRAS (121P), and KIT (1PKG). Discovery Studio Visualizer was used to visualize the genes, and PyMOL created the complex.

#### 3.7.3 Structural retrieval of compounds (PubChem)

The PDB format is used to retrieve the structures of the bacterial compounds from PubChem discovered by GC-MS analysis described in Table 2.

#### 3.7.4 Prediction of target genes according to retrieved metabolites (g: Profiler)

Gene enrichment analysis were done through g: profiler, and the target genes were TP53, PTEN, MSH6, APC, CHEK2, PMS2, KRAS, BR1P1, CDKN2A, MET, EPCAM, CTNNB, BRAF, MSH2, BARD1, RB1, MUTYH, TERT, HRAS, and KIT (Supplementary Figure S1).

#### 3.7.5 Protein–protein network construction (STRING)

**FIGURE 9:**
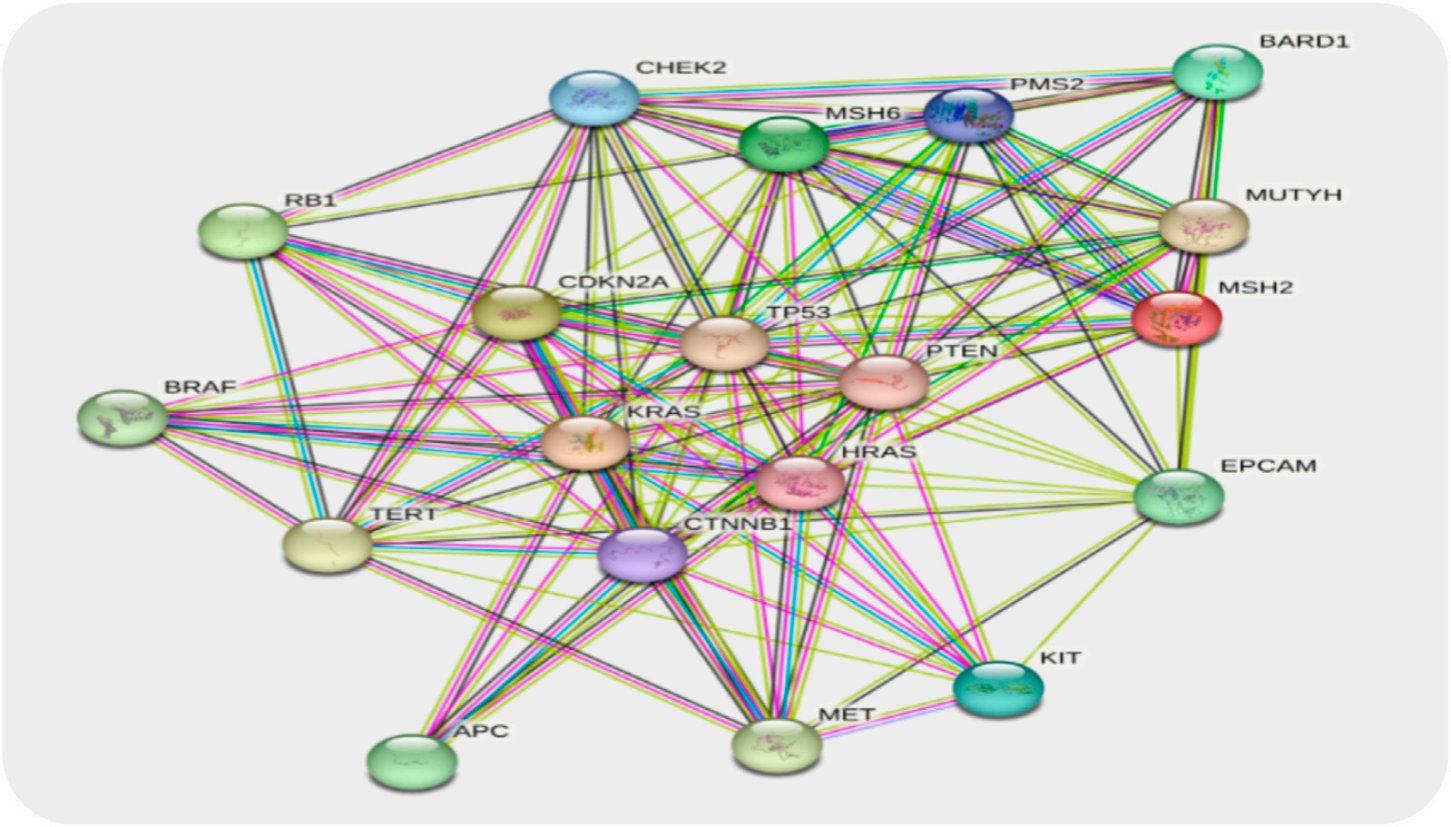
PPI network of epithelial glioblastoma cancer genes. Each node represents the relevant gene, and the edges represent protein–protein associations.

PPI networks are constructed by hiding nodes connected to independent targets and using a confidence level greater than 0.40. In the PPI network, protein–protein interactions are shown as edges and proteins are shown as nodes. The network has 19 nodes, 119 edges, and an average node-to-node distance of 12.5° (Figure 9).

#### 3.7.6 Molecular docking analysis (PyRx)

**FIGURE 10:**
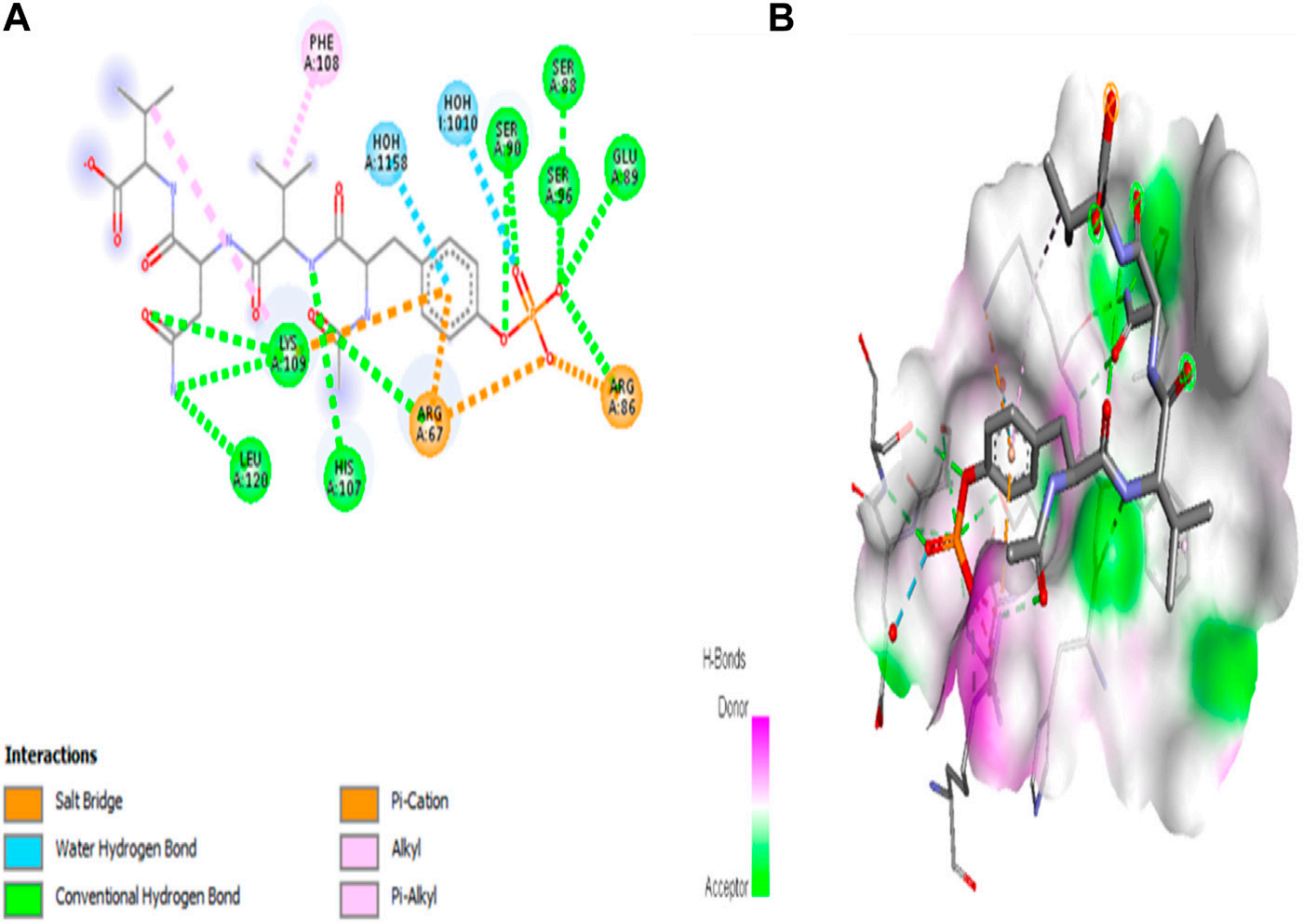
Molecular interaction of 1, 2-benzenedicarboxylic acid and MET (−7.9). **(A)** 2D image of the docked complex; **(B)** 3D image of the docked complex.

With all targeted genes or docking scores, five compounds were molecularly docked. When compared to all other substances, 1, 2-benzenedicarboxylic acid has a high docking energy, with gene MET being −7.9 (Table 3) (Figure 10).

**TABLE 3:** Binding energies of docked complexes of identified compounds and cancerous genes.

Cancerous genes	Binding energies (KJ/mol)
MET	−7.9
EPCAM	−7.2
KIT	−6.9
KRAS	−6.9
PTEN	−6.8
BRAF	−6.6
CTNNB	−6.4
CHEK2	−6.8
MUTYH	−6.2
MSH2	−5.9
RB1	−5.8
BR1P1	−5.7
TP53	−5.7
PMS2	−5.6
MSH6	−5.4
APC	−5.3
CDKN2A	−5.1
BARD1	−5.1
HRAS	−5
TERT	−4.9

#### 3.7.7 Pharmacokinetic study (ADME)

**FIGURE 11:**
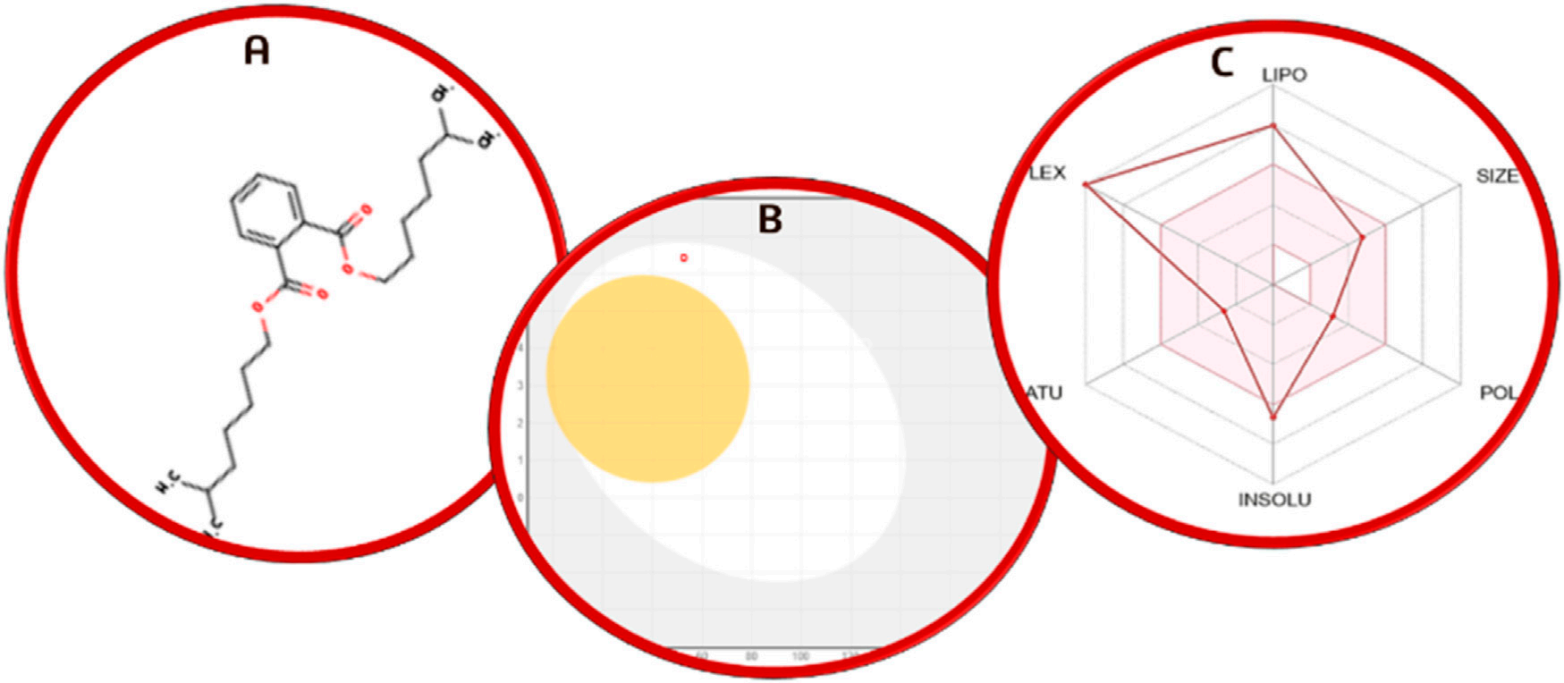
**(A)** Chemical structure prediction of 1, 2-benzenedicarboxylic acid; **(B)** BOILED-Egg representation; **(C)** radar plot of 1,2-benzenedicarboxylic acid.

Swiss ADME results showed that 1, 2-benzenedicarboxylic acid is the most effective anti-cancer compound against epithelial glioblastoma cancer genes due to its high lipophilicity. The LogP value of the compound is greater than 4 which means it is water soluble. C_24_H_38_O_4_ is the molecular formula of diethylhexylphthalate, and the molecular weight is 390.56 s g/mol. The drug was effectively absorbed in the GI tract, according to the pharmacokinetic analysis, and it did not break Lipinski’s rule. The compounds’ interactions and pharmacokinetic analyses revealed low glycoprotein permeability and no inhibitory effect on CYP2C19 and CYP2C9. Figure 11 shows a BOILED-Egg representation of 1, 2-benzenedicarboxylic acid.

#### 3.7.8 Venn analysis (bioinformatics and evolutionary genomics analysis)

**FIGURE 12:**
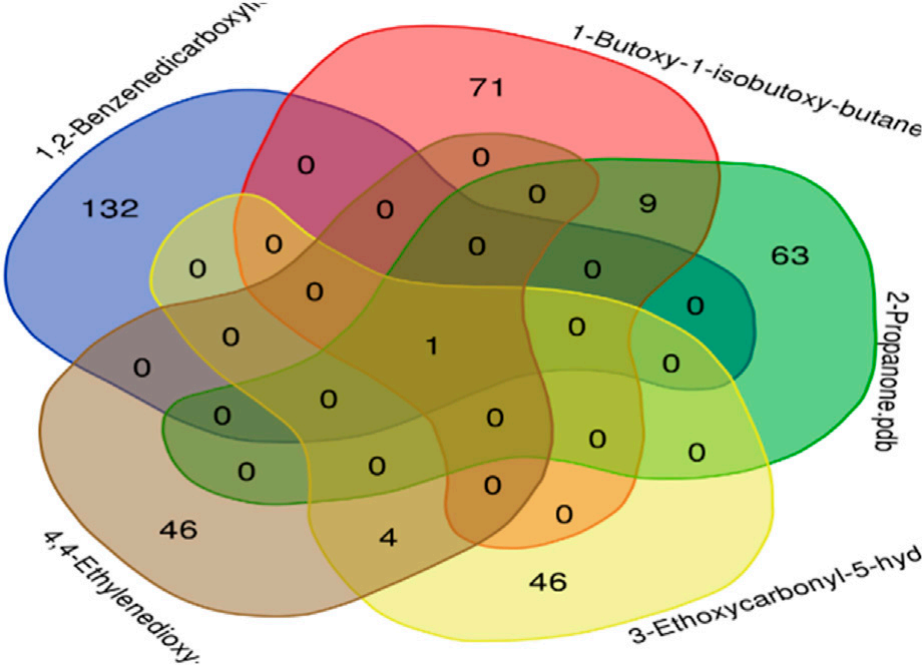
A Venn diagram illustrating the intersection of identified compounds with the following elements: 1,2-benzenedicarboxylic acid (132), 1-butoxy-1-isobutoxy-butane (71), 2-propanone (63), 3-ethoxycarbonyl-5-hydroxytetrahydropyran-2-one (46), and 4,4-ethylenedioxyl-1-pentylamine.

The intersection of recognized targets about identified chemical compounds and epithelial glioblastoma cancer is depicted in the Venn diagram. A total of five compounds are obtained using 1,2-benzenedicarboxylic acid, 1-butoxy-1-isobutoxy-butane, 2-propanone, 3-ethoxycarbonyl-5-hydroxytetrahydropyran-2-one, and 4,4-ethylenedioxy-1-pentylamine to create Venn diagrams (Figure 12).

#### 3.7.9 Molecular dynamic simulation (iMODs)

**FIGURE 13:**
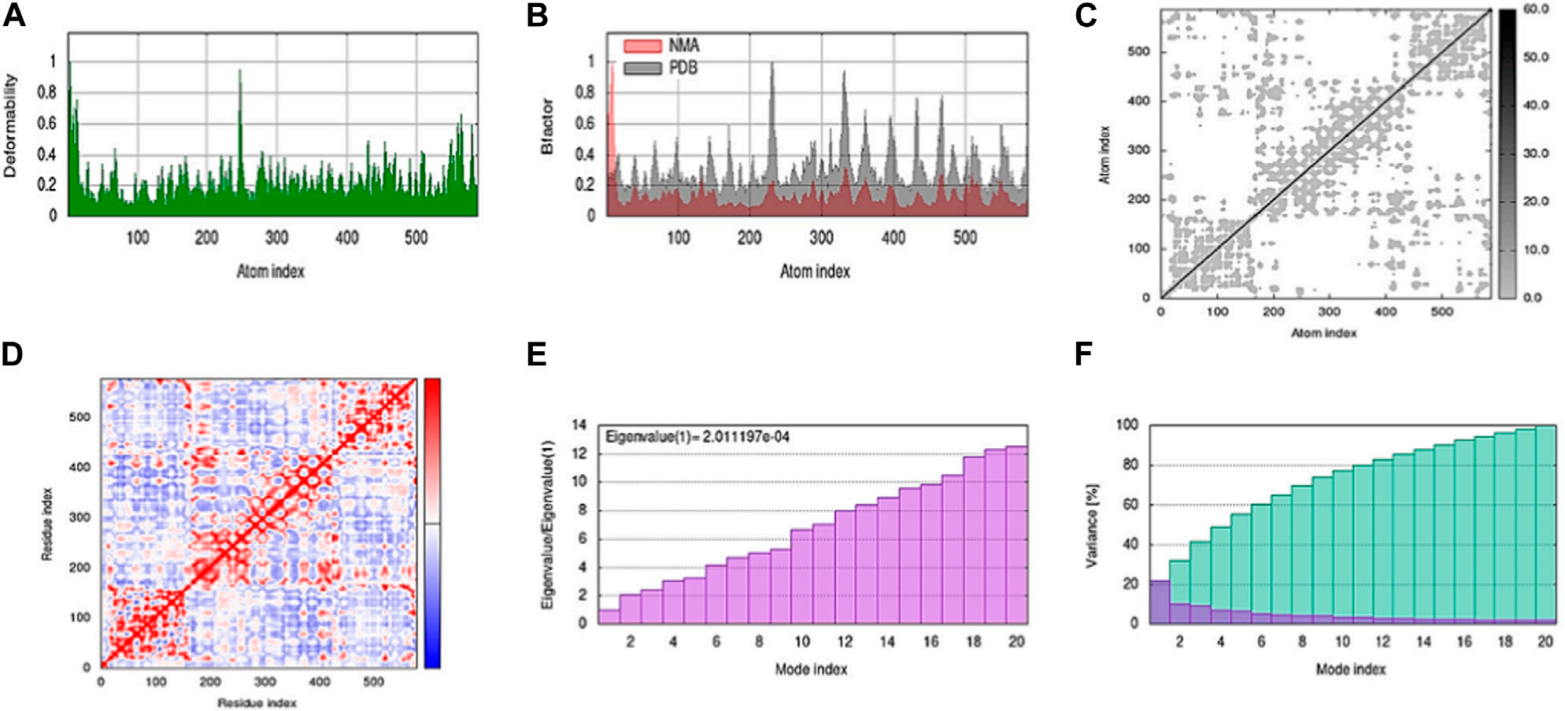
Shows a simulation of molecular dynamics of the best docked complex. **(A)** Deformability of the complex; **(B)** B-factor graph; **(C)** elastic network (gray matter indicates a stiffer region); **(D)** covariance map: correlated (red), uncorrelated (white), or anti-correlated (blue) motions; **(E)** The eigenvalue plot illustrates the minimum energy required to deform the complex; **(F)** Variance individual variance (purple) and cumulative variance (green).

MD stimulation was performed on the iMOD server. The docked complex was used to perform the MD stimulation. Results from iMODs, including deformability, variance, eigenvalue, covariance map, elastic network, and B-factor, are shown in Figure 13.

#### 3.7.10 QSAR modeling (3D-QSAR)

**FIGURE 14:**
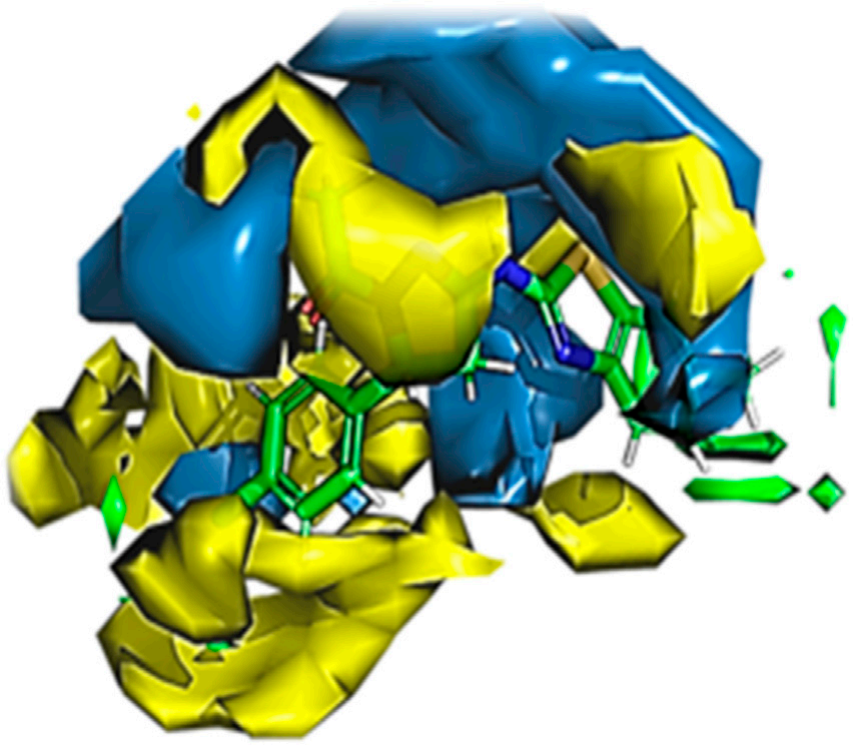
Contour diagram of the best hit compound, 1,2-benzenedicarboxylic acid.

The *in silico* 3D-QSAR research’s objective is to evaluate how the structural characteristics of the targeted compounds impacted their biological activities. In order to determine the biological activities of the ligands, the method primarily makes use of three-dimensional properties. A dataset of five compounds derived through GC-MS analysis was used to construct the QSAR model. The best ligand’s activity was predicted by the generated model, which demonstrated its accuracy. Predicting which ligand would be the most active was a way to test the model. For 1,2-benzenedicarboxylic acid, a good statistic was obtained in all models. The significant r2 and the cross-validated correlation coefficient q2 were both 0.1712. Figure 14 shows the contour map for the compound with the best hit.

## 4 Discussion

The *Bacillus* genus is best known for producing some of the most well-known natural products due to its extremely active metabolism. A number of significant metabolites are produced by *Bacillus* species, such as iturin, surfactin, fengycin, and cyclic lipopeptide antibiotics. Surfactin, streptavidin, and bacillomycin D are some of these natural therapies that can be used to treat cancer (Zhou et al., 2018). They belong to the *Bacillus* family, which has been shown to produce a spectrum of secondary metabolites, such as polyketides (PKs), terpenes, and siderophores, as well as ribosomally and non-ribosomally produced peptides with anti-cancer properties (Harwood et al., 2018). However, further study is needed to uncover the potential biological functions of other elements.

One of the most intriguing subgroups of natural product producers is the *Bacillus* genus, and studies on it frequently result in the discovery of new active compounds (Mondol et al., 2013). The *Bacillus* genus has also been demonstrated to be a significant producer of novel antibiotics. *Bacillus* metabolites are widely distributed and have a variety of bioactivities. On the basis of recent research, we still know very little about the metabolite repertoire of *Bacillus*. *Bacillus* metabolites are thought to be a source of potential new drugs that require further investigation, despite the fact that some have been shown to inhibit bacteria and suppress cancer (Shao et al., 2021).

In this study, *Bacillus* strains were collected from the Molecular Biology and Biotechnology culture collection Laboratory (MBBL) and were identified through 16S rRNA PCR amplification. The DNA was extracted using the CTAB procedure and visualized through gel electrophoresis. After successful PCR amplification of the 16S rRNA gene, the strain was identified and verified through GenBank submission (Hassan, 2016). Following the identification, *Bacillus* spp. strains were cultured, and metabolites were extracted from the culture medium. Then, these extracts were subjected to GC–MS analysis. GC-MS revealed the presence of 29 compounds, with major compounds including 1,2-benzenedicarboxylic acid, 1,1-butoxy-1-isobutoxy-butane, 2-propanone, 3,3-ethoxycarbonyl-5-hydroxytetrahydropyran-2-one, and 4,4-ethylenedioxy-1-pentylamine (Nas et al., 2021). Previous research findings, such as those of Munjal et al. (2016), have identified similar compounds from *Bacillus* strains using GC-MS. The majority of bacterial secondary metabolites are known for their diverse characteristics, including antioxidant, anti-inflammatory, and antimicrobial properties (Munjal et al., 2016). The anti-inflammatory and antioxidant potential of these metabolites was demonstrated through protein denaturation inhibition and DPPH analysis, respectively. Additionally, their antimicrobial activity was compared with that of cefoxitin, yielding promising results (Gao et al., 2018).

This study extended into the realm of cancer research. Target genes for epithelial glioblastoma cancer were identified, and ligands such as 1,2-benzenedicarboxylic acid were docked against these genes. Among the ligands, this compound (1,2-benzenedicarboxylic acid) exhibited the highest binding affinity, particularly with crucial cancer-related genes, including MET, EPCAM, PTEN, and CHEK2. 3D-QSAR analysis confirmed its promising characteristics, and ADMET analysis indicated its suitability for drug development. This study underscores the untapped potential of *Bacillus* metabolites and their diverse applications, from antimicrobial and anti-inflammatory agents to potential anticancer drugs. The compound 1,2-benzenedicarboxylic acid emerges as a strong candidate for further exploration in cancer treatment, with the potential to effectively target a variety of cancer cell lines.

## 5 Conclusion

According to the study, the bacterial metabolites present in *Bacillus* bacteria have antioxidant, antibacterial, anti-hemolytic, anti-inflammatory, anti-diabetic, and anti-cancerous activity *in vitro*. The GC-MS analysis of the bacterial extract revealed the discovery of several bacterial compounds with substantial biological activity. Bacterial compounds derived from these fractions could be potential therapeutic candidates. The compounds identified as ligands through GC-MS analysis were evaluated *in silico* against epithelial glioblastoma cancer genes, with the results indicating that 1,2-benzenedicarboxylic acid is a strong candidate for new therapeutics because of its good ADMET properties and high binding energy properties. These findings suggest that *Bacillus* species are a promising treatment option for inflammation and other human-related illnesses. The medicinal or pharmacological potential of the *Bacillus* spp. revealed that it is quite promising as a versatile therapeutic bacterium and that more research should be done on it. These compounds have not previously been reported from *Bacillus* spp., according to the literature review. While little work has been done to use these compounds, fundamental research should be enhanced in order to leverage the hidden potentials of both these compounds and the organisms that make them, as this could lead to uncovering of an entirely novel world of metabolism in bacteria.

## Data Availability

The datasets presented in this study can be found in online repositories. The names of the repository/repositories and accession number(s) can be found in the article/Supplementary Material.
