# Supplementary material for: GC–MS profiling of Bacillus spp. metabolites with an in vitro biological activity assessment and computational analysis of their impact on epithelial glioblastoma cancer genes
Source: Front Chem. 2023 Dec 5;11:1287599. doi: 10.3389/fchem.2023.1287599 (PMC10728721; doi:10.3389/fchem.2023.1287599)
Supplement: Supplementary file 1 [file Image1.pdf]

(A). (B). (C).

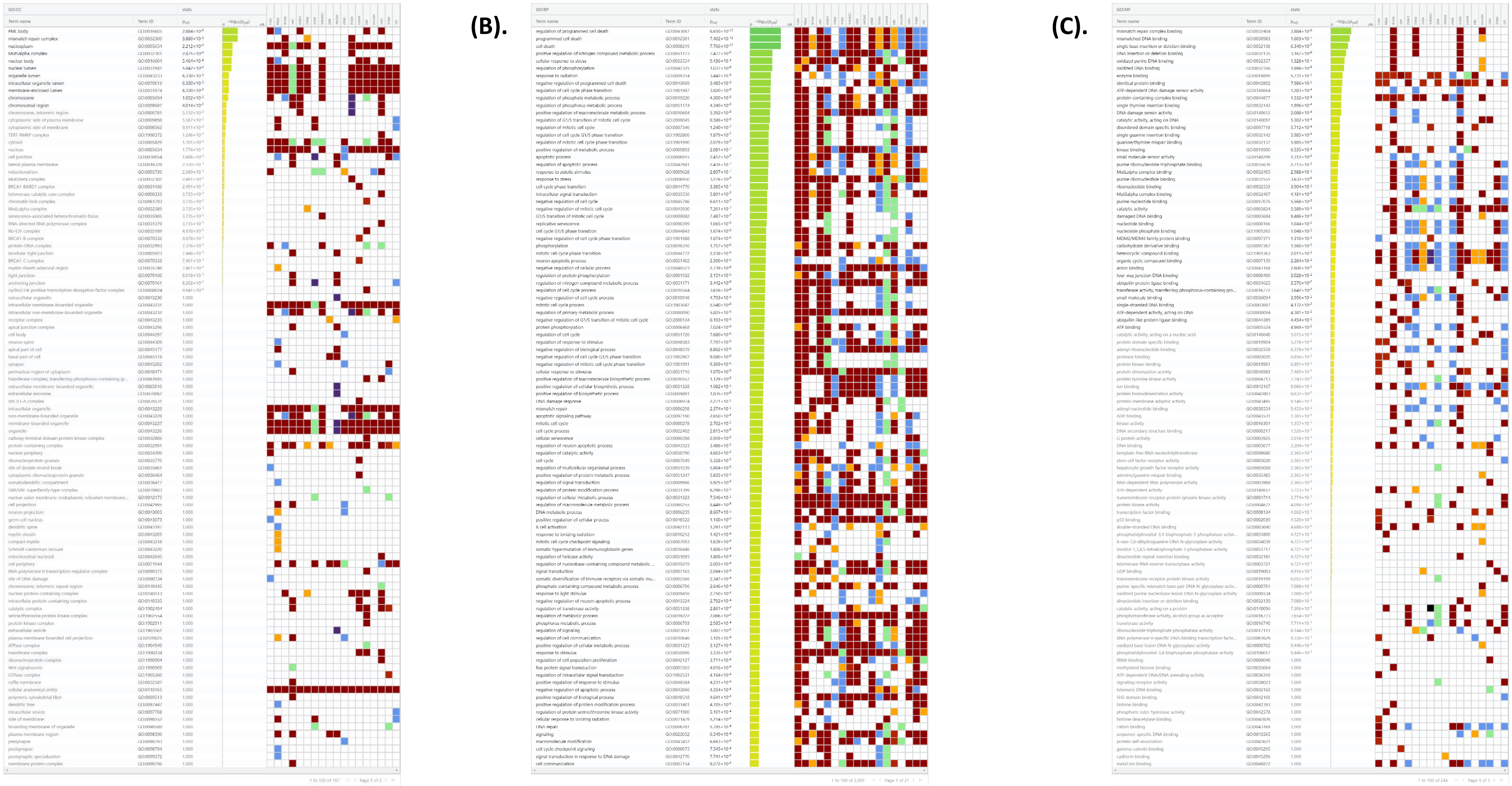

Figure S1: Gene enrichment analysis of genes (A). Cellular Process (B). Biological process (C). Molecular function.
